# Improving diagnostic accuracy of blood culture-positive cases in a cancer center via an antimicrobial stewardship program and infectious disease consultations

**DOI:** 10.1038/s41598-024-53543-w

**Published:** 2024-02-04

**Authors:** Naoya Itoh, Nana Akazawa, Takanori Kawabata, Makoto Yamaguchi, Eiichi N. Kodama, Norio Ohmagari

**Affiliations:** 1 Division of Infectious Diseases, Aichi Cancer Center Hospital, Nagoya, Japan; 2 Collaborative Chairs Emerging and Reemerging Infectious Diseases, National Center for Global Health and Medicine, Graduate School of Medicine, Tohoku University, Miyagi, Japan; 3 Department of Data Science, National Cerebral and Cardiovascular Center, Suita, Japan; 4 Division of Infectious Diseases, International Research Institute of Disaster Science, and Graduate School of Medicine, Tohoku University and Tohoku Medical Megabank Organization, Sendai, Japan; 5 AMR Clinical Reference Center, Disease Control and Prevention Center, National Center for Global Health and Medicine, Tokyo, Japan; 6 Disease Control and Prevention Center, National Center for Global Health and Medicine, Tokyo, Japan

**Keywords:** Infectious diseases, Oncology, Epidemiology

## Abstract

The direct impact of antimicrobial stewardship programs (ASP) and infectious disease (ID) consultations on patients' clinical diagnoses remains unknown. We assessed their influence on improving the diagnostic accuracy of blood culture-positive inpatients at a Japanese cancer center. Our single-center, retrospective observational study was conducted from April 1, 2018 to March 31, 2022 to evaluate two phases: pre-intervention (notification of antimicrobials by the infection control team) and post-intervention (ASP implementation and ID consultation service establishment). There were 42,514 inpatients: 22,096 during the pre-intervention and 20,418 during the intervention periods. A total of 939 blood culture-positive episodes (pre-intervention, n = 434; post-intervention, n = 505) were analyzed. During the pre-intervention period, 28.1% of the patients had an unknown diagnosis, which decreased significantly to 1.2% post-intervention. Furthermore, hepatobiliary tract and other infections increased significantly post-intervention, and the mortality rate due to *Staphylococcus aureus* infection decreased from 28.6% pre-intervention to 10.4% post-intervention. The trend and level of the total number of culture specimens submitted per 1000 patient days for all culture specimens increased significantly post-intervention. Notably, the two-set rate of monthly blood cultures increased significantly. In conclusion, improving the overall diagnostic process with ASP and ID consultations at cancer centers could lead to the optimization of patient care.

## Introduction

Antimicrobial stewardship programs (ASP) play a vital role in healthcare by providing multiple benefits, such as reducing inappropriate antimicrobial use, lowering antimicrobial costs, decreasing the emergence of antimicrobial-resistant bacteria, and preventing drug-related adverse events^1–3^. Similarly, infectious disease (ID) consultation services have been shown to enhance clinical outcomes in patients with specific infections and mitigate the prevalence of drug-resistant organisms^4–7^. Despite these known benefits, the direct impact of ASP and ID consultations on enhancing patient diagnosis remains unknown. Notably, an accurate diagnosis is paramount as it dictates the targeted and effective treatment pathway. Diagnostic stewardship (DS) aims to optimize patient care by improving the overall diagnostic process. However, the intervention measures involve ordering, processing, and reporting of diagnostic tests, which differ from the approaches of ASP and ID consultations^8–12^.

Patients with cancer often undergo multiple courses of antimicrobial therapy because of its prophylactic use, the occurrence of febrile neutropenia during cancer treatment, or the emergence of serious infections as the underlying disease progresses^1^. Infections that arise during cancer treatment may disturb planned surgeries or chemotherapy, thereby worsening the prognosis. Consequently, ASP and ID consultations play a vital role in guiding appropriate antimicrobial therapy for patients with cancer, as infections are markedly more common in this group than in other populations and settings. In a previous study conducted at a cancer center in Japan, we found that ASP and ID consultation services curtailed the use of broad-spectrum antimicrobials and reduced certain drug-resistant organisms without compromising patient outcomes^1^. Interestingly, although many community hospitals in Japan have general internal medicine physicians to handle intricate diagnostic challenges, the situation differs in cancer centers, where ID specialists are relied on to diagnose both infectious and noninfectious diseases^13^. The precise contribution of ASP and diagnostic support provided by ID specialists, especially in the context of the diagnostic process, remains unclear.

Patients with bloodstream infections have a high mortality rate, and appropriate diagnostic and management strategies are necessary^14^. Therefore, the support of a specialized ID team for such patients is deemed highly essential. In this study, we aimed to evaluate the impact of ASP and ID consultation services on improving the diagnostic process of blood culture-positive patients at a Japanese cancer center.

## Methods

### Setting

This study was conducted at the Aichi Cancer Center (ACC), a 500-bed tertiary care facility in Aichi, Japan. The center has 23 clinical departments and admits approximately 11,000 patients annually. Specifically, 15 departments are in charge of inpatients: plastic and reconstructive surgery, hematology and cell therapy, thoracic surgery, thoracic oncology, gastroenterological surgery, gastroenterology, orthopedic surgery, head and neck surgery, breast oncology, neurosurgery, urology, gynecological oncology, radiation oncology, diagnostic and interventional radiology, and clinical oncology. The Antimicrobial Stewardship Team (AST) comprises one ID specialist (increased to two from April 1, 2021), a pharmacist, two laboratory technicians, and an infection control nurse.

### Study design

This was a single-institution retrospective observational study conducted over 48 months between April 1, 2018, and March 31, 2022.

### Interventions

The interventions evaluated in this study were implemented in two phases:

1. Phase 1: Pre-intervention period (April 1, 2018 to March 31, 2020).
2. Phase 2: Implementation of ASP and establishment of ID consultation service (April 1, 2020 to March 31, 2022).

### Inclusion criteria

The inclusion criteria were all inpatients with positive blood cultures between April 1, 2018 and March 31, 2022.

### ASP

On weekdays, ID specialists promptly communicated positive blood culture results to the primary physician team to ensure appropriate empiric therapy. Over the weekend, the laboratory or ID specialists did not communicate positive blood culture results to the primary physician team but reported them the following weekday. In cases where *Staphylococcus aureus* (*S. aureus*) or *Candida* spp. were detected, an ID consultation was suggested and the patients were examined at the bedside.

Moreover, audits were conducted thrice weekly at the AST conferences for all patients on (i) specific anti-methicillin-resistant *S. aureus* agents—vancomycin, teicoplanin, daptomycin, linezolid; and (ii) particular broad-spectrum antibiotics—cefepime, cefozopran, piperacillin–tazobactam, imipenem–cilastatin, meropenem, and doripenem. Patients who had already received an ID consultation were excluded from the audit. For AST conferences, the pharmacist retrieved data of patients who received specific antimicrobials, from ACC’s electronic medical record system. The laboratory technicians shared the latest microbiological information pertaining to these patients with the other AST members. Upon identifying drug-resistant organisms in the patient, the infection control nurse quickly alerted the manager of the ward where the patient was admitted and provided necessary infection control measures. ID specialists oversaw the comprehensive management of each professional's role in this process. All cases reviewed at the conferences were documented in the electronic medical record. For patients judged by the AST as requiring either modification of antimicrobial therapy or further additional culture tests due to insufficient testing, feedback was provided to the primary physician team on the audit day, either by phone call or a note in the patients’ medical record. All audited patients were monitored daily on weekdays until the completion of their specific antimicrobial therapy or for the duration of follow-up required by the AST. The AST contacted the primary physician teams whenever necessary. From April 2021, the audits were extended to all weekdays, and fluoroquinolones were added to the targeted antimicrobial list. For patients who were deemed to require bedside evaluation by the ID specialists due to challenges in diagnosing and determining treatment for IDs based solely on review of medical record information by the AST conference, the ID specialists contacted the primary physician team to propose an ID consultation.

### ID consultation service

Dedicated ID specialists provided patient consultation from primary physician teams in the 15 aforementioned departments on all weekdays.

### Data collection

For all patients with positive blood cultures, the following information was collected from ACC’s electronic medical record system for each episode: age, sex, department, date of blood culture, fluid collection, microorganism detection, diagnosis, 30-day all-cause mortality, and whether the ID specialists provided feedback. The ACC database provided information regarding the number of specimen submissions, AS reviews, and ID consultations. We accessed these data on August 5, 2023 for research purposes.

### Definition

The diagnosis was determined from medical record descriptions, and missing descriptions were categorized as unknown. When the infection focus was clear, the diagnosis was not classified as febrile neutropenia, regardless of the presence of neutropenia. The term “multiple infection” was defined as the presence of two distinct ID conditions without a clear determination of which caused the bacteremia. The two-set blood culture rate was defined as (number of two sets/total sets) × 100, while the blood culture positivity rate was defined as (number of positive sets/total sets) × 100. Positive blood culture episodes with unknown outcome were excluded from the calculation of all-cause 30-day mortality. Respiratory specimens included sputum, pharyngeal mucus, nasal mucus, oral mucus, lung tissue, and bronchial lavage fluid. Gastrointestinal specimens encompassed stool, bile, and pancreatic fluid. Genitourinary specimens consisted of urine and vaginal secretions. Puncture fluid specimens comprised pleural fluid, ascitic fluid, spinal fluid, joint fluid, and bone marrow fluid. Other materials included catheter tips and wound cultures, drain cultures, and cultures obtained from various sources.

### Primary outcome measures

The primary outcome presented the proportion of patients with an unknown diagnosis documented in the medical record per positive blood culture.

### Secondary outcome measures

Secondary outcomes assessing the impact of ASP and ID consultations on diagnostic testing included the following: (i) number of each type of culture specimen submitted per 1000 patient-days of hospitalization, (ii) blood culture two-set rate, and (iii) blood culture positivity rate. Culture specimens for inpatients included blood, respiratory, gastrointestinal, and genitourinary specimens, puncture fluid, other materials, and total specimens. Culture specimens used for screening were excluded from the study. Moreover, to determine the prognostic impact of our intervention, we evaluated the all-cause 30-day mortality rate of patients with blood culture-positive episodes. Second and subsequent episodes of bacteremia in the same patient during the study period were included in the mortality rate. In addition, the mortality rate included all deaths, including those caused by cancer and IDs.

### Statistical analyses

Pearson’s Chi-square and Fisher’s exact tests were used for categorical variables, while the Wilcoxon rank-sum test was used to analyze continuous variables. The primary endpoints and 30-day mortality rates were compared between the pre- and post-intervention periods (April 1, 2018 to March 31, 2020 and April 1, 2020 to March 31, 2022, respectively). The secondary endpoints other than the 30-day mortality rate were examined using an interrupted time series. Due to the exploratory nature of this study, no adjustments were made for multiple comparisons. A p value less than 0.05 was considered statistically significant. Analyses were performed using the R software, version 4.2.0 (The R Foundation for Statistical Computing, Vienna, Austria) and SPSS version 28 (IBM Corp., Armonk, NY, USA).

### Ethical considerations

This study was approved by the Institutional Review Board of the ACC Hospital (approval number: 2023-0-161) and was conducted according to the principles of the Declaration of Helsinki. The requirement for informed consent was waived by the Institutional Review Board of ACC Hospital because this study only used data collected in clinical practice.

## Results

### Patient admissions and interventions

During the study period, 42,514 patients were admitted to the ACC, with 22,096 and 20,418 patients in the baseline and intervention periods, respectively. Throughout the intervention period, the AST provided 1438 feedbacks, with 1376 ID consultations. The average acceptance rate of proposals from the ASTs was 80.8% (range 70.6–100%); specifically, for those with *S. aureus* bacteremia and candidemia including polymicrobial bacteremia episodes, the acceptance rates were 68/70 (97.1%) and 17/17 (100.0%), respectively.

### Blood culture-positive episode characteristics

A total of 939 blood culture-positive episodes (pre-intervention, n = 434; post-intervention, n = 505) were included in the study. All patients with positive blood culture results in the pre- and post-intervention periods were admitted to the hospital.

Specifically, in the post-intervention period, four people died before the assessment, resulting in 501/505 (99.2%) intervention episodes. Patient characteristics of blood culture-positive episodes pre- and post-intervention are shown in Table 1. Pre- and post-intervention, the average age was 65.9 and 67.0 years, respectively (p = 0.10). Moreover, the number of male patients was 270 (62.2%) and 317 (62.8%) pre- and post-intervention, respectively (p = 0.31). Furthermore, post-intervention, there were fewer positive blood culture episodes in the Hematology and Cell Therapy and Orthopedic Surgery departments than pre-intervention (p < 0.01, p = 0.04, respectively); however, there were no significant differences in the other departments pre- and post-intervention. Isolated microorganisms in culture-positive blood episodes pre- and post-intervention are shown in Table 2.

**Table 1:** Baseline characteristics of blood culture-positive episodes pre- and post-intervention.

	Before n = 434	After n = 505	p value
Sex, male	270 (62.2%)	317 (62.8%)	0.31
Age, years (mean ± SD)	65.9 ± 12.3	67.0 ± 12.1	0.10
Medical department			
Breast oncology	7 (1.6%)	16 (3.2%)	0.12
Clinical oncology	113 (26.0%)	130 (25.7%)	0.92
Diagnostic and interventional radiology	6 (1.4%)	6 (1.2%)	0.79
Gastroenterological surgery	92 (21.2%)	108 (21.4%)	0.94
Gastroenterology	98 (22.6%)	131 (25.9%)	0.23
Gynecologic oncology	6 (1.4%)	16 (3.2%)	0.07
Head and neck surgery	16 (3.7%)	28 (5.5)	0.18
Hematology and cell therapy	54 (12.4%)	33 (6.5%)	< 0.01
Neurosurgery	1 (0.2%)	1 (0.2%)	0.71
Orthopedic surgery	12 (2.8%)	5 (1.0%)	0.04
Plastic and reconstructive surgery	1 (0.2%)	0 (0.0%)	0.46
Radiation oncology	5 (1.2%)	2 (0.4%)	0.18
Thoracic oncology	12 (2.8%)	16 (3.2%)	0.72
Thoracic surgery	2 (0.5%)	1 (0.2%)	0.44
Urology	9 (2.1%)	12 (2.4%)	0.76

*SD* standard deviation.

Data are expressed as numbers (percentages) or as mean ± SD where indicated.

**Table 2:** Bacteria isolated from blood culture-positive episodes pre- and post-intervention.

	Before n = 434	After n = 505	p value
Gram-positive			
*Bacillus* spp.	6 (1.4%)	8 (1.6%)	0.80
*Corynebacterium* spp.	6 (1.4%)	4 (0.8%)	0.53
MRSA	12 (2.8%)	20 (4.0%)	0.31
MSSA	24 (5.5%)	47 (9.3%)	0.029
Other *Staphylococcus*	82 (18.9%)	51 (10.1%)	< 0.001
*Enterococcus* spp.	38 (8.8%)	39 (7.7%)	0.57
*Streptococcus* spp.	26 (6.0%)	28 (5.5%)	0.77
*Propionibacterium* spp.^a^	7 (1.6%)	5 (1.0%)	0.39
GP-Others	19 (4.4%)^c^	10 (2.0%)^d^	0.28
Gram-negative			
*Acinetobacter* spp.	4 (0.9%)	7 (1.4%)	0.51
Aeromonas spp.	5 (1.2%)	2 (0.4%)	0.26
*Bacteroides* spp.	10 (2.3%)	6 (1.2%)	0.19
*Escherichia coli*, non-ESBL	33 (7.6%)	51 (10.1%)	0.18
*Escherichia coli*, ESBL	18 (4.1%)	25 (5.0%)	0.56
*Klebsiella* spp., non-ESBL	24 (5.5%)	56 (11.1%)	0.002
*Klebsiella* spp., ESBL	2 (0.5%)	3 (0.5%)	1.00
*Enterobacter* spp.^b^	20 (4.6%)	17 (3.4%)	0.33
*Citrobacter* spp.	5 (1.2%)	3 (0.6%)	0.48
*Serratia* spp.	6 (1.4%)	6 (1.2%)	0.79
*Pseudomonas aeruginosa*	12 (2.8%)	13 (2.6%)	0.86
GN-Others	14 (3.2%)^e^	20 (4.0%)^f^	0.55
Fungus			
*Candida* spp.	16 (3.7%)	17 (3.4%)	0.80
Other-fungus	3 (0.7%)^g^	2 (12.5%)^h^	0.67
Polymicrobial bacteria	42 (9.7%)	63 (12.9%)	0.13

Data are expressed as numbers (percentages).

^a^*Propionibacterium* spp. includes *Cutibacterium* spp.

^b^*Enterobacter* spp. includes *Enterobacter aerogenes.*

^c^GP-Others includes six unidentifiable Gram positive rods, three *Micrococcus* spp., two each of *Eggerthella lenta* (*E. lenta*) and *Parvimonas micra* (*P. micra*), and one each of *Anaerococcus prevotii*, *Bifidobacterium* species, *Clostridium perfringens*, *Granulicatella adiacens* (*G. adiacens*), *Peptococcus anaerobius* and *Peptoniphilus asaccharolyticus.*

^d^GP-Others includes two *P. micra* and one each of unidentifiable Gram positive rod, *Gemella bergeri*, *Actinomyces odontolyticus*, *G. adiacens*, *E. lenta*, *Ruminococcus gnavus*, *Bifidobacterium longum,* and *Gordonia sputi.*

^e^GN-Others includes four *Raoultella planticola*, three *Morganella morganii*, two each of *Proteus mirabilis* and *Veillonella* spp., and one each of unidentifiable anaerobic gram-negative bacilli, *Mycoplasma hominis,* and *Prevotella oralis.*

^f^GN-Others includes four *Proteus* spp., three *Raoultella* spp., two each of *Prevotella* spp. and *Campylobacter* spp., and one each of *Achromobacter denitrificans*, *Aggregatibacter aphrophilus*, *Dialister pneumosintes*, *Edwardsiella tarda*, *Fusobacterium nucleatum*, *Haemophilus influenzae*, *Pandoraea apista*, *Parabacteroides distasonis,* and *Stenotrophomonas maltophilia.*

^g^Other-fungus includes three *Exophiala dermatitidis* (*E. dermatitidis*).

^h^Other-fungus includes two *E. dermatitidis.*

*ESBL* extended-spectrum β-lactamase, *GN* gram-negative, *GP* gram-positive, *MRSA* methicillin-resistant *Staphylococcus aureus*, *MSSA* methicillin-susceptible *Staphylococcus aureus.*

Furthermore, populations of methicillin-susceptible *S. aureus* and extended-spectrum β-lactamase non-producing *Klebsiella* spp. were higher post-intervention (p = 0.029, p = 0.002, respectively), unlike those of *Staphylococcus* spp., which were lower (p < 0.001). Other microorganisms exhibited no significant differences pre- and post-intervention.

Table 3 details the diagnoses of culture-positive blood episodes for both periods. Notably, the proportion of unknown diagnoses decreased significantly from 28.1% pre-intervention to 1.2% post-intervention (p < 0.01). Hepatobiliary tract infections and other types of infections increased significantly post-intervention (p < 0.001 and p < 0.01, respectively).

**Table 3:** Comparison of clinical diagnoses of positive blood culture episodes pre- and post-intervention.

	Before n = 434	After n = 505	p value
Specific diagnosis available	312 (72.0%)	499 (98.8%)	< 0.001
Hepatobiliary tract infections	76 (17.5%)	169 (33.5%)	< 0.001
Intra-abdominal infections	25 (5.8%)	32 (6.3%)	0.71
Genitourinary infections	59 (13.6%)	84 (16.6%)	0.20
CRBSI	72 (16.6%)	104 (20.6%)	0.12
Respiratory infections	13 (3.0%)	16 (3.2%)	0.88
Skin and soft tissue infections	4 (0.9%)	5 (1.0%)	1.00
Bone and joint infections	3 (0.7%)	9 (1.8%)	0.40
Gastrointestinal infection	0 (0.0%)	2 (0.4%)	0.50
Febrile neutropenia	25 (5.8%)	22 (4.4%)	0.33
CNS infections	0 (0.0%)	1 (0.2%)	1.00
Cardiovascular infections	1 (0.2%)	0 (0.0%)	0.46
Intra-tumor infections	5 (1.2%)	5 (1.0%)	0.81
Multiple infections	0 (0.0%)	3 (0.6%)^a^	0.25
Others	4 (0.9%)^b^	18 (3.6%)^c^	< 0.01
Contamination	24 (5.5%)	29 (5.7%)	0.89
Diagnosis unknown	122 (28.1%)	6 (1.2%)	< 0.01

Data are expressed as numbers (percentages).

^a^Multiple infections included one case each of CRBSI with liver abscess, CRBSI with iliopsoas abscess, and cholangitis with pelvic abscess.

^b^Each included one case of bacteremia due to skin barrier failure, one case of bacterial translocation, one case of iliopsoas abscess, and one case of submandibular sialadenitis.

^c^Each included 10 cases of bacteremia from the tumor and skin-mucosa barrier failure, four cases of bacterial translocation, one case of bacterial parotitis, one case of cervical abscess, one case of posterior pharyngeal abscess, and one case of mediastinal abscess.

*CNS* central nervous system, *CRBSI* catheter-related bloodstream infection, *Multiple infections* presence of multiple pathologies causing bacteremia simultaneously.

Table 4 presents the all-cause 30-day mortality rates for each isolated microorganism in culture-positive blood episodes. Specifically, the mortality rate for *S. aureus* declined significantly from 28.6% pre-intervention to 10.4% post-intervention (p = 0.02). No significant differences were observed in the mortality rates of culture-positive episodes among all microorganisms and other microorganisms.

**Table 4:** Comparison of the 30-day all-cause mortality of blood culture-positive episodes pre- and post-intervention.

	Before (%)	After (%)	p value
All isolated bacteria	72/434 (16.7)	81/505 (16.0%)	0.77
*Staphylococcus aureus*	10/35 (28.6%)	7/67 (10.4%)	0.02
Other* Staphylococcus*	12/81 (14.8%)	3/51 (5.8%)	0.16
Enterococcus spp.	8/38 (21.1%)	5/39 (12.8%)	0.34
*Candida* spp.	5/16 (31.3%)	9/17 (52.9%)	0.21
Enterobacterales	15/115 (13.0%)	26/168 (15.5%)	0.20
Polybacteremia	11/42 (26.2%)	17/63 (27.0%)	0.93

Data are expressed as numbers (percentages).

### Culture specimen submissions

**Figure 1:**
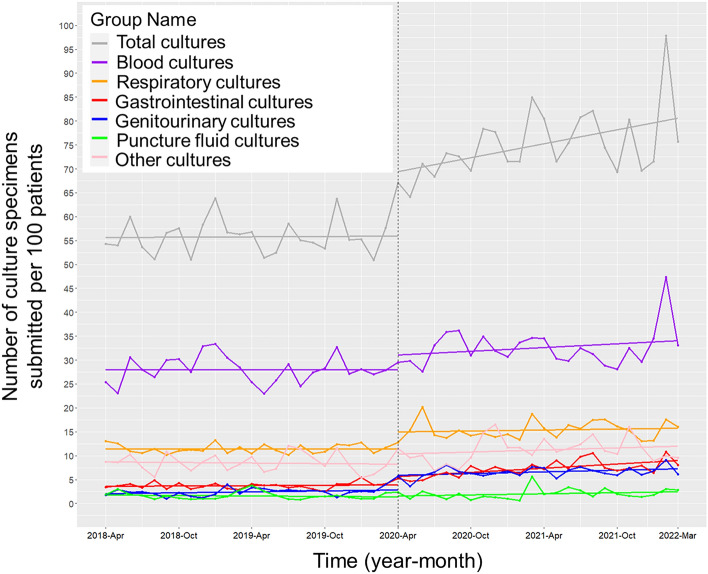
Trends in the number of culture specimens submitted per 1000 patients, by month, during the pre-intervention period (Phase 1) and interventional period (Phase 2). Each dot signifies the number of culture specimens submitted per 1000 patients each month, and the slope is calculated based on linear regression in two phases. Specifically, Phase 1 refers to the pre-intervention period from April 1, 2018 to March 31, 2020. Phase 2, in contrast, covers the implementation of the ASP and the establishment of the ID consultation service from April 1, 2020 to March 31, 2022. The analysis yielded the following results: (1) Total cultures: Both the monthly trend and levels demonstrated a significant increase (coefficient for trend 0.47; 95% CI 0.05 to 0.90, p < 0.03; coefficient for levels 13.47; 95% CI 7.60 to 19.34, p < 0.001); (2) Blood cultures: Neither the trend nor levels exhibited a significant change (coefficient for trend 0.13; 95% CI − 0.15 to 0.41, p = 0.37; coefficient for levels 3.03; 95% CI − 0.91 to 6.97, p = 0.14); (3) Respiratory cultures: Although no significant trend increase was observed (coefficient 3.59; 95% CI − 0.10 to 0.16, p = 0.58), a significant level increase was noted (coefficient 3.50; 95% CI 1.76 to 5.23, p < 0.001); (4) Gastrointestinal cultures: Both the monthly trend and levels showed significant increases (coefficient for trend 0.14; 95% CI 0.05 to 0.22, p = 0.002; coefficient for levels 1.71; 95% CI 0.58 to 2.83, p = 0.005); (5) Genitourinary cultures: While no significant trend increase was found (coefficient 0.02; 95% CI − 0.05 to 0.10, p = 0.52), a significant level increase was documented (coefficient 3.05; 95% CI 2.04 to 4.06, p < 0.001); (6) Puncture fluid cultures: Neither the trend nor levels displayed a significant change (coefficient for trend 0.06; 95% CI − 0.02 to 0.13, p = 0.17; coefficient for levels 0.14; 95% CI − 0.93 to 1.21, p = 0.80); (7) Other cultures: Neither the trend nor levels demonstrated a significant change (coefficient for trend 0.09; 95% CI − 0.09 to 0.28, p = 0.33; coefficient for levels 2.04; 95% CI − 0.50 to 4.59, p = 0.12).

During the post-intervention period, there was a significant increase in the trend and level of monthly culture specimens per 1000 patients for both total cultures (trend coefficient 0.47; 95% confidence interval [CI] 0.05 to 0.90, p < 0.03; level coefficient 13.47; 95% CI 7.60 to 19.34, p < 0.001) and gastrointestinal cultures (trend coefficient 0.14; 95% CI 0.05 to 0.22, p = 0.002; level coefficient 1.71; 95% CI 0.58 to 2.83, p = 0.005) (Fig. 1). The monthly trends for respiratory and genitourinary culture specimens per 1000 patients did not increase, but their levels did rise significantly (respiratory culture coefficient 3.50; 95% CI 1.76 to 5.23, p < 0.001; genitourinary culture coefficient 3.05; 95% CI 2.04 to 4.06, p < 0.001). There were no significant changes in the monthly trends or levels for blood cultures, and puncture fluid cultures per 1000 patients.

### Blood culture metrics

**Figure 2:**
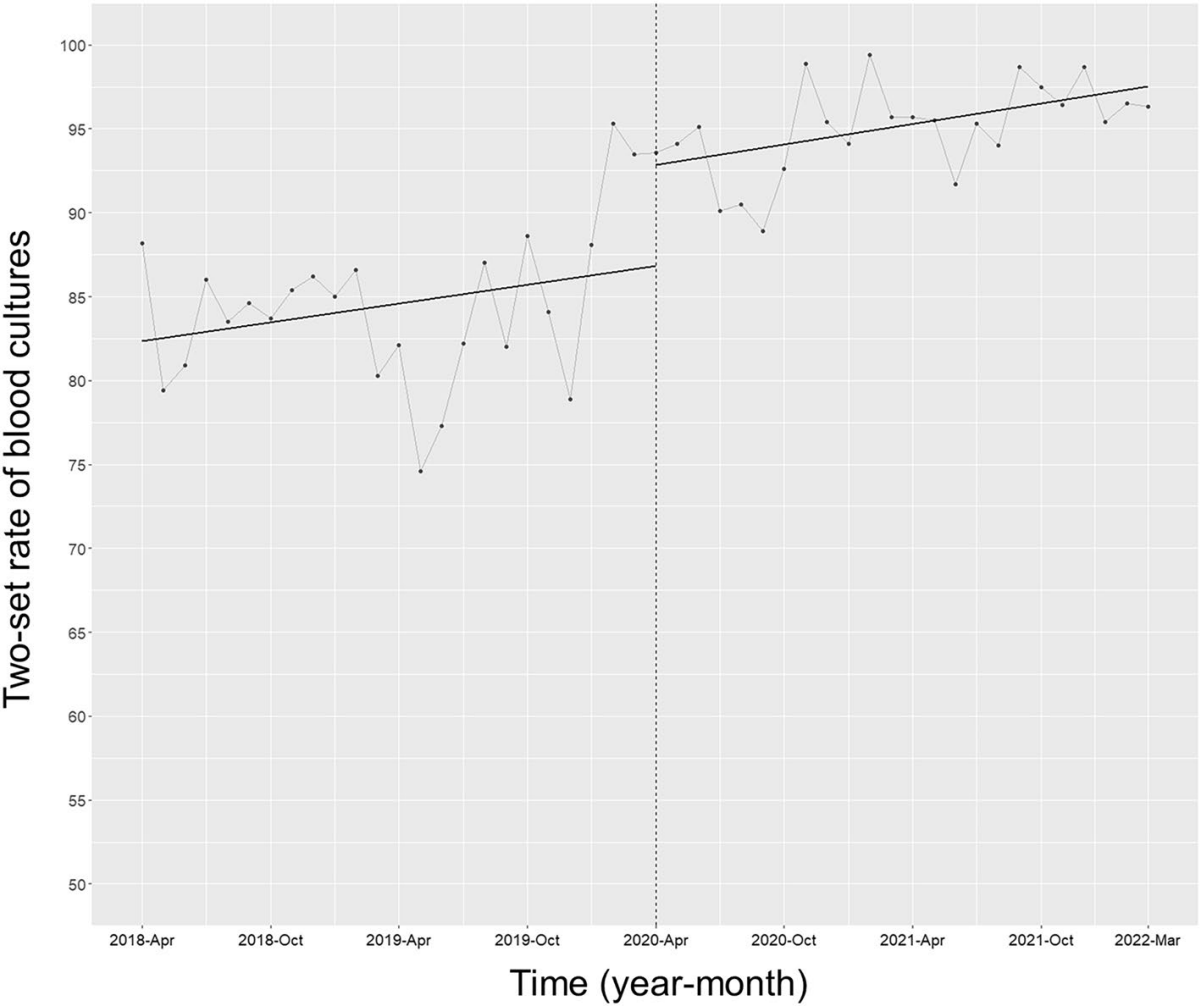
Trends in the two-set rate of blood cultures, by month, during the pre-intervention period (Phase 1) and interventional period (Phase 2). Each dot in the graph signifies the two-set rate of blood cultures each month, with the slope calculated based on linear regression for the two phases. Specifically, Phase 1 encompasses the pre-intervention period from April 1, 2018 to March 31, 2020, while Phase 2 refers to the period from April 1, 2020 to March 31, 2022, during which the ASP was implemented and the ID consultation service was established. Importantly, the trend in the two-set rate of monthly blood cultures did not show a significant increase following the intervention (coefficient − 0.02, 95% CI − 0.29 to 0.32, p = 0.92). However, the level did exhibit a significant rise (coefficient 6.05, 95% CI 1.84 to 10.25, p = 0.007).

**Figure 3:**
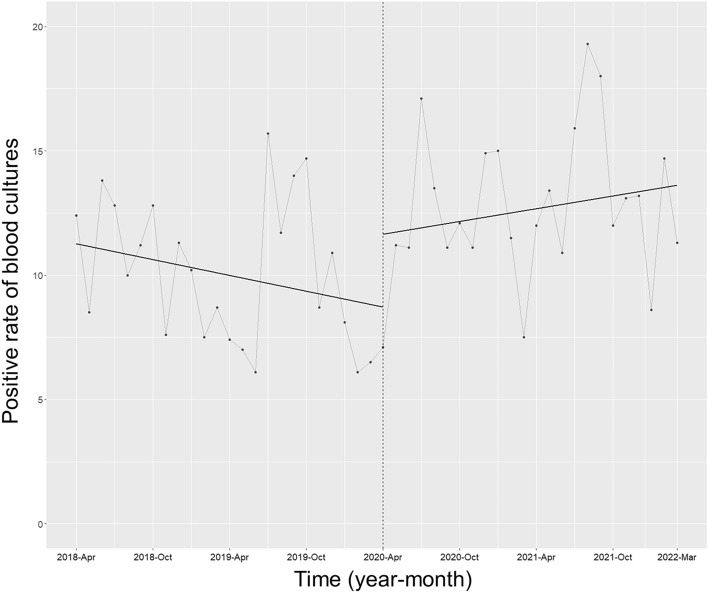
Trends in the positive rate of blood cultures, by month, during the pre-intervention period (Phase 1) and interventional period (Phase 2). Each dot on the graph represents the positive rate of blood cultures each month, and the slope is determined based on linear regression across the two phases. Specifically, Phase 1 refers to the pre-intervention period from April 1, 2018 to March 31, 2020, while Phase 2 entails the implementation of the ASP and the establishment of the ID consultation service from April 1, 2020 to March 31, 2022. Importantly, neither the trend in the positive rate of blood cultures (coefficient 0.19; 95% CI − 0.05 to 0.43, p = 0.13) nor its level (coefficient 2.91; 95% CI −  0.42 to 6.25, p = 0.09) showed a significant change following the intervention.

The two-set rate of blood cultures was 84.3 ± 4.6 and 95.0 ± 2.7 pre- and post-intervention, respectively. The trend in the two-set rate of monthly blood cultures did not increase post-intervention, but the level did significantly (coefficient 6.05; 95% CI 1.84 to 10.25, p = 0.007) (Fig. 2). Moreover, the rate of positive blood cultures was 10.2 ± 2.8 pre-intervention and increased to 12.7 ± 3.0 post-intervention. There was no significant change in the positive blood culture rate trend or its levels (Fig. 3).

## Discussion

This is the first study to evaluate the diagnostic improvements with ASP and ID consultations in a Japanese cancer center. Our main finding was an evident enhancement in the diagnostic process of blood culture-positive patients’ post-intervention, potentially improving patient care.

In our study, we observed a significant decrease in the proportion of patients with an unknown diagnosis, accompanied by an increase in hepatobiliary tract infections and other types of infections. This finding could be attributed to the early detection of bacteremia cases and appropriate intervention by the AST, as well as its collaboration with ID specialists, who provided more personalized care to patients who could not be adequately evaluated through AST conferences alone. Considering that a standard treatment duration has been established for each type of infection^15^, patients should receive appropriate treatment once the origin of the bacteremia is determined. Notably, in the clinical diagnosis of blood culture-positive episodes after intervention, 10 of 18 cases of bacteremia due to tumor and skin-mucosa barrier failure were classified under the “others” category. Cancer chemotherapy, radiation therapy, surgical procedures, medical devices, and local tumors disrupt normal anatomical barriers, such as skin and mucosal surfaces, allowing external microorganisms to invade the human body^16^. Such conditions could not be identified without meticulous physical examination and medical interviews, underscoring the challenges of diagnosing certain infections based solely on descriptions in electronic medical records, emphasizing the crucial role of bedside evaluation by ID specialists for patients with positive blood culture results.

Although there was no overall reduction in mortality after interventions for blood culture-positive episodes, there was a significant reduction in mortality associated with *S. aureus* infections. Previous studies have reported reduced mortality due to *S. aureus* bacteremia and candidemia after ID consultation^6,17^, which is attributed to adherence to the bundle through ID consultation, emphasizing appropriate treatment duration, timely rechecking of blood cultures and echocardiography, and catheter removal. Furthermore, decreased mortality rates have been reported for *Candida* spp., gram-negative bacilli, and enterococci; however, no significant changes were observed^6,18–20^. The implications of our study’s results may be limited due to the small sample size of microorganisms and a lack of detailed information on individual patient severity, underlying diseases, and comorbidities. Furthermore, considering the increase in the number of blood culture samples submitted during the post-intervention period, it is possible that some patients with bacteremia may have died without being diagnosed during the pre-intervention period. The significant reduction in mortality from *S. aureus* bacteremia and decreasing trend in mortality from other staphylococcal and enterococcal bacteremia indicate that our intervention did not worsen outcomes. Importantly, a proper diagnosis can lead to improvements in the overall quality of care.

Here, the level of the two-set rate of blood cultures increased post-intervention. Specifically, collecting blood cultures in two sets is recommended to enhance detection sensitivity and determine contamination when the results are positive^21^. Therefore, increasing the two-set rate post-intervention is viewed as a positive outcome. Although there was no significant increase in the number of blood cultures, an increasing trend was observed. The positivity rate of appropriate blood cultures ranged from 5 to 15%^22^ and that of blood cultures post-intervention was 12.7%, suggesting that the change in the number of blood culture tests associated with the intervention was appropriate and not due to over-testing.

We observed a significant increase in the total number of culture samples, including respiratory, gastrointestinal, and genitourinary cultures, following the intervention in our study. DS aims to reduce unnecessary testing and false-positive results, rapidly identify pathogens, tailor treatments, and guide appropriate clinical behavior^23^. Although we did not directly evaluate the quality of testing during our intervention, it is worth noting that ID specialists recommended collection of the necessary specimens on a case-by-case basis. Considering the decrease in undiagnosed positive blood culture episodes post-intervention and the satisfactory positive rate of blood cultures, there is likely an increase in the submission of appropriate specimens rather than excessive testing. Furthermore, in our previous study on antimicrobial use at the same institution and within the same time period, we observed a decrease in the use of broad-spectrum antimicrobials and an increase in narrow-spectrum antimicrobials 1 year post-intervention^1^. This finding suggests that appropriate de-escalation was performed based on culture results, correlating with the increased number of specimens submitted. To further enhance the quality of clinical practice, it is essential to promote DS in patients throughout the hospital in conjunction with ASP and ID consultations.

This study had some limitations. First, as the research was conducted at a single Japanese cancer center, the generalizability of our intervention remains uncertain. Second, diagnoses were based on descriptions in medical records, which could potentially obscure the true significance of positive blood culture episodes. Third, the mortality rates per bacterial species were not adjusted for confounding factors, as information on individual patient characteristics was not collected.

In conclusion, ASP and ID consultations at a cancer center significantly reduced the proportion of unexplained causes among blood culture-positive patients without negatively affecting patient outcomes. Furthermore, the number of culture specimens required for diagnosis and treatment post-intervention increased. Our intervention suggests that improving the overall diagnostic process could lead to the optimization of patient care.

## Data Availability

The datasets used and/or analyzed in the current study are available from the corresponding author upon request.
